# Carriers of Loss-of-Function Mutations in *EXT* Display Impaired Pancreatic Beta-Cell Reserve Due to Smaller Pancreas Volume

**DOI:** 10.1371/journal.pone.0115662

**Published:** 2014-12-26

**Authors:** Sophie J. Bernelot Moens, Hans L. Mooij, H . Carlijne Hassing, Janine K. Kruit, Julia J. Witjes, Michiel A. J. van de Sande, Aart J. Nederveen, Ding Xu, Geesje M. Dallinga-Thie, Jeffrey D. Esko, Erik S. G. Stroes, Max Nieuwdorp

**Affiliations:** 1 Department of Vascular Medicine, Academic Medical Center, Amsterdam, the Netherlands; 2 Department of Paediatrics, University of Groningen, University Medical Center Groningen, Groningen, the Netherlands; 3 Department of Orthopaedics, LUMC, Leiden, the Netherlands; 4 Department of Radiology, Academic Medical Center, Amsterdam, the Netherlands; 5 Department of Cellular and Molecular Medicine, UC San Diego, San Diego, California, United States of America; 6 Department of Experimental Vascular Medicine, Academic Medical Center, Amsterdam, the Netherlands; University of Lille Nord de France, France

## Abstract

Exotosin (EXT) proteins are involved in the chain elongation step of heparan sulfate (HS) biosynthesis, which is intricately involved in organ development. Loss of function mutations (LOF) in *EXT1* and *EXT2* result in hereditary exostoses (HME). Interestingly, HS plays a role in pancreas development and beta-cell function, and genetic variations in *EXT*2 are associated with an increased risk for type 2 diabetes mellitus. We hypothesized that loss of function of *EXT1* or *EXT2* in subjects with hereditary multiple exostoses (HME) affects pancreatic insulin secretion capacity and development. We performed an oral glucose tolerance test (OGTT) followed by hyperglycemic clamps to investigate first-phase glucose-stimulated insulin secretion (GSIS) in HME patients and age and gender matched non-affected relatives. Pancreas volume was assessed with magnetic resonance imaging (MRI). OGTT did not reveal significant differences in glucose disposal, but there was a markedly lower GSIS in HME subjects during hyperglycemic clamp (iAUC HME: 0.72 [0.46–1.16] vs. controls 1.53 [0.69–3.36] nmol·l^−1^·min^−1^, p<0.05). Maximal insulin response following arginine challenge was also significantly attenuated (iAUC HME: 7.14 [4.22–10.5] vs. controls 10.2 [7.91–12.70] nmol·l^−1^·min^−1^ p<0.05), indicative of an impaired beta-cell reserve. MRI revealed a significantly smaller pancreatic volume in HME subjects (HME: 72.0±15.8 vs. controls 96.5±26.0 cm^3^ p = 0.04). In conclusion, loss of function of EXT proteins may affect beta-cell mass and insulin secretion capacity in humans, and render subjects at a higher risk of developing type 2 diabetes when exposed to environmental risk factors.

## Introduction

Heparan sulfate proteoglycans (HSPGs) play a role in many biological processes including fine-tuning most of the physiological and pathological processes related to fetal organ development, lipid metabolism and inflammation [1]. *EXT1* and *EXT2* genes encode for an endoplasmic reticulum-resident glycosyltransferase complex involved in chain elongation and possibly chain initiation of heparan sulfate biosynthesis [2], [3]. The *EXT* gene family consists of 5 genes, including *EXTL1* (EXT-like 1), *EXTL2* and *EXTL3*, which encode proteins that catalyze GlcNac transferase reactions. Whereas the function of EXT1 and EXT2 has been widely recognized, the precise role of EXTL3 and a related protein EXTL2 in heparan sulfate (HS) biosynthesis remains unclear [4]–[6]. Of note, Extl3 was reported to be involved in murine pancreatic beta-cell development [7]–[9].

Heterozygous loss of function (LOF) mutations in *EXT1* and *EXT2* are known to be involved in the development of hereditary multiple exostoses (HME) syndrome [10], a disorder with a reported prevalence of 1/50.000 individuals [11], and have been shown to lead to both locally (exostosis plate) [12] and systemically [13] altered heparan sulfate composition. Consequently, growth of multiple bony tumors (i.e. exostoses or osteochondromas) after birth and throughout childhood, lasting until closure of the growth plates, were observed, which can result in skeletal deformities and malignancies [14]. Main complications are a direct result of compression of neighbouring tissue or structures and involve pain, disturbance of blood circulation, and in rare cases spinal/cervical cord compression [15].

Interestingly, common single nucleotide polymorphisms (SNPs) in *EXT*2 were associated with increased risk for the development of type 2 diabetes mellitus (DM2) [16]. Despite conflicting results [17]–[21], a recent meta-analysis of Liu et al replicated the original observed significant association between common genetic variants in *EXT*2 and the risk of developing DM2 [22]. In line, SNPs in *EXT*2 have also been associated with impaired glucose clearance in DM2 as assessed by an oral glucose tolerance test [23]. Of note, both *EXT1* and *EXT2* genes are expressed in human pancreas (https://www.lsbm.org) pathophysiological role of *EXT* in pancreas function (insulin secretion) remains to be elucidated.

In the present study we designed a dedicated series of investigations to unravel the effect of disrupted heparan sulfate synthesis on beta-cell function and mass, as well as insulin secretion, in humans with heterozygous loss of function mutations in *EXT1* or *EXT2*.

## Methods

We enrolled Dutch HME subjects, based on established heterozygous mutations in either *EXT1* or *EXT2*, and non-carrier relatives over 18 years of age, without pre-existent type 1 or 2 diabetes. We tested for alterations in glucose metabolism and beta-cell reserve. Written informed consent was obtained after explanation of the study. The study was approved by the institutional review board of the Academic Medical Center of the University of Amsterdam and carried out according to the Declaration of Helsinki.

### Oral Glucose Tolerance Test (OGTT) and hyperglycemic clamp

After an overnight fast, a standardized OGTT was performed. After baseline venous sampling subjects were asked to ingest 75 g glucose. At t = 30, 60, 90 and 120 minutes a 4.5 ml blood sample was obtained for assessment of blood glucose, insulin and C-peptide.

On a separate study day, a hyperglycemic clamp was performed after an overnight fast. On the day of study antecubital veins of both arms were cannulated for blood sampling and infusion of fluids. All bedside glucose measurements were performed using a calibrated glucose sensor (YSI 2300 STAT S; YSI, Yellow Springs, OH). Based on the fasting plasma glucose level and the subject's bodyweight first phase insulin secretion was determined using a 20% glucose bolus (weight/70×10 – plasma glucose  =  ml required) given over 1 min, aiming to achieve a plasma glucose level of 14 mmol/l. Subsequently, blood glucose levels were maintained at 14 mmol/l by continuous glucose infusion. Pump settings (glucose infusion rate) were adapted based on blood glucose levels at t = 0, 2.5, 5, 7.5, 10 and 20 minutes. Simultaneously, blood samples were collected for insulin and C-peptide determination. After 120 minutes an arginine bolus (5 gram) was given, followed by measurement of plasma insulin levels at t = 125, 130,140 and 150 minutes. Basal fasting glucose, HbA1c, total cholesterol, HDL and LDL cholesterol, triglycerides and free fatty acids (FFAs) were assessed in fasting plasma using standard laboratory procedures. GLP-1 levels were determined using al RIA_LINCO_ assay [24]. Osteocalcin was measured using an immunoradiometric assay (Biosource/Medgenix Diagnostics, Fleuris, Belgium) as previously published [25]. Fecal elastase levels were assessed using a commercially available ELISA kit for Elastase 1 (ScheBo).

Glucose was determined by the hexokinase method (Hitachi), Insulin was determined on an Immulite 2000 system (Diagnostic Products, Los Angeles, CA). C-peptide was measured by RIA (RIA-coat C-peptide; Byk-Sangtec Diagnostica, Dietzenbach, Germany).

Using data from the OGTT and clamp, homeostasis model assessment (HOMA) indexes were calculated for insulin sensitivity (HOMA-ir  =  insulin (picomoles)/6.945*glucose (millimoles)/22.5) and insulin secretion (HOMA-β  = 20* fasting insulin (picomoles)/6.954/glucose (millimoles-3.5). Under stable conditions of constant hyperglycemiathe amount of glucose infused (milligrams per kilogram) equals the amount of metabolized glucose (M). M was calculated as the average glucose infusion rate during the last 30 min of the clamp (*t* = 90 through *t* = 120). The M value divided by the average plasma insulin concentration (I) during the same interval, the M/I ratio, provides a measurement of tissue sensitivity to insulin (micrograms per kilogram per minute per picomole per liter) [26]. The disposition index is calculated as the product of the M/I ratio. Insulin sensitivity was estimated, using the metabolic clearance rate (MCR) of glucose and the insulin sensitivity index (ISI), as described previously [27]. Overall glucose-stimulated insulin secretion was calculated as AUC_insulin_/AUC_glucose_ ratio.

### Magnetic resonance imaging

In a subset of previous participants (both HME-subjects and healthy controls) we performed abdominal imaging preceding the OGTT, using a 3-T MR scanner (Intera, Philips Healthcare, Best, The Netherlands). A T2-weighted two-dimensional transversal half-Fourier single-shot turbo spin-echo (HASTE) sequence was obtained in a breath hold to determine the volume of the pancreas. Scan parameters were: TR/TE 600/70 ms; FA 90 degrees; number of slices: 20, FOV450 mm×315 mm, voxel sizes: 1.4 mm×1.4 mm×4.00 mm; slice gap 1 mm. Images were analyzed by 2 independent, blinded investigators (ICC = 0.85, 95% CI 0.61–0.94) using ITK Snap software version 2.4 (University of Pennsylvania). Pancreatic area was delineated in each imaging slice and the number of voxels in this area was determined, subsequently this number was transcribed to volume in cubic millimetres. The mean area of separate measurements was used.

### Power calculation and statistical analysis

Based on our previous findings in a oral glucose tolerance tests in carriers with a rare loss-of function mutation (ABCA1) [28], the difference between the mean incremental area under the curve (IAUC) for the carriers (245 [153–353]) and the controls (155 [84–198]), was 90 mmol l^−1^ min^−1^. Assuming a two group t-test with a 0,050 two-sided significance level and a power of 80%, we need to include 14 subjects in each group.

Data are presented as mean ± SD or medians with interquartile range [IQR] unless stated otherwise. Normally distributed baseline characteristics were compared using a student's *t* test (all but triglycerides). Differences in triglyceride and free fatty acid levels (FFA), known not to be normally distributed, and continues outcome variables were assessed using the nonparametric Mann-Whitney U test. All repeated measurements are reported by incremental AUC (area above baseline), computed by the trapezoidal rule. A p-value of less than 0.05 was used to indicate significant differences. All analyses were performed with SPSS software version 19.0.0.1.

## Results

### Beta-cell function and glucose metabolism in human EXT carriers versus controls

**Figure 1:**
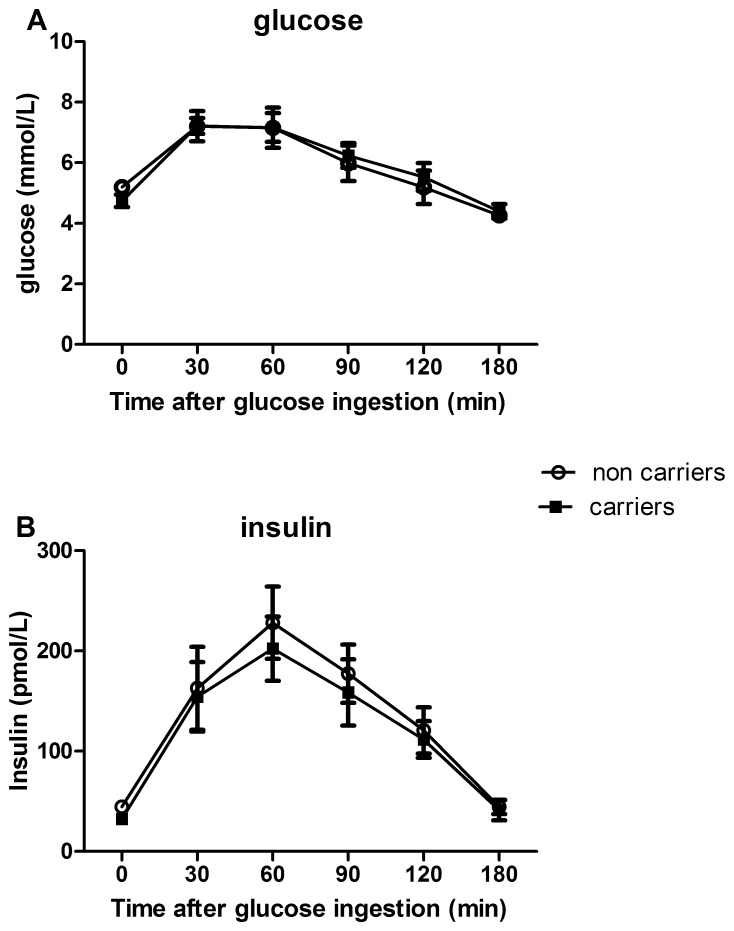
Plasma glucose (A) and insulin curves (B) after OGTT in HME subjects (closed squares) and controls (circles).

We included 16 *EXT1* carriers and 6 *EXT2* carriers (for a list of identified mutations see S1 and S2 Table) as well as 26 age and gender matched non carrier controls, whom participated in the OGTT, clamp or both (for baseline characteristics per study see S3 and S4 Tables). Age, BMI, fasting glucose, HbA1C and insulin levels, as well as basal lipid levels (including FFAs – only in OGTT group, see S3 Table), were all comparable between carriers and control subjects (Table 1). HME subjects are characterized by elevated osteocalcin levels (S3 Table), a protein recognized as a marker of bone formation [29]. Assessing exocrine pancreas function by fecal elastase [30], no differences were found (S3 Table). No difference was reported in family history for diabetes (Table 1). *EXT* carriers and controls displayed a similar response during OGTT with respect to plasma glucose (iAUC: carriers: 233 [157–286] vs controls: 160 [100–281] nmol·l^−1^·min^−1^ n.s.) and plasma insulin levels (iAUC: carriers; 17.4 [6.5–24.1] vs controls; 18.3 [12.6–23.0] nmol·l^−1^·min^−1^) (Figs. 1A and 1B). Markers of insulin resistance and beta-cell function were not significantly different between the HME subjects and controls (Table 2).

**Table 1:** Baseline characteristics of all study subjects.

	Noncarriers	Carriers	P-value
	(*N = 25)*	*(N = 22)*	
Age (years)	45±14	38±10	0.064
Men	11 (42)	7 (31)	
BMI	25.0±3.30	25.5±4.3	0.591
BSA	1.9±0.21	1.9±0.19	0.505
Cholesterol (mmol/l)			
Total	5.26±1.25	4.87±1.16	0.295
LDL	3.23±1.14	3.03±1.06	0.537
HDL	1.51±0.41	1.34±0.41	0.153
Triglycerides (mmol/l)	0.90[0.67–1.19]	0.87[0.55–1.34]	1.00
Fasting glucose (mmol/l)	5.0±0.66	4.8±0.48	0.251
Hba1c			
mmol/mol	36±3.9	34±3.6	0.403
%	5.4±0.34	5.3±0.32	0.391
Fasting insulin (pmol/l)	49±34	39±28	0.291
Family history			
Diabetes	*4 (15*	*3 (14)*	
CVD	*3 (12)*	*4 (29)*	

Data are means ± SD, *n (%)*, or median [IQR]. Abbreviations: BMI  =  Body Mass Index; BSA  =  Body Surface Area; LDL  =  Low Density Lipoprotein. HDL  =  High Density Lipoprotein. CVD  =  Cardiovascular Disease.

**Table 2:** Beta-cell function and insulin sensitivity parameters.

	Noncarriers	Carriers	P-value
Baseline HOMA index (all)			
HOMA-ir	1.23[0.80–1.80]	0.95[0.56–1.47]	0.16
HOMA-β	79[48–128]	78[49–147]	0.89
Insulinogenic index (ogtt)	50.7[37.8–176.5]	60.3[31.1–71.4]	1.00
(pmol/mmol)			
AUC_insulin_/AUC_glucose_ ratio	87.8[73.6–261.4]	79.8[50.6–105.2]	1.00
(ogtt) (pmol/mmol)			
ISIcomp (ogtt)	23.9[20.9–41.5]	33.5[18.5–46.9]	0.07
(µmol/(kg min pmol L))			
Disposition index (clamp)	22.1[15.2–41.9]	25.6[10.0–33.1]	1.00
MCR (ogtt) (ml/(min kg))	9.8[9.1–10.4]	10.1[9.2–10.6]	0.37

Values are presented as median [interquartile range]. Abbreviations: HOMA  =  homeostatis model assessment; AUC  =  area under the curve; ISIcomp  =  index of composite whole-body insulin sensitivity; MCR  =  metabolic clearance rate.

**Figure 2:**
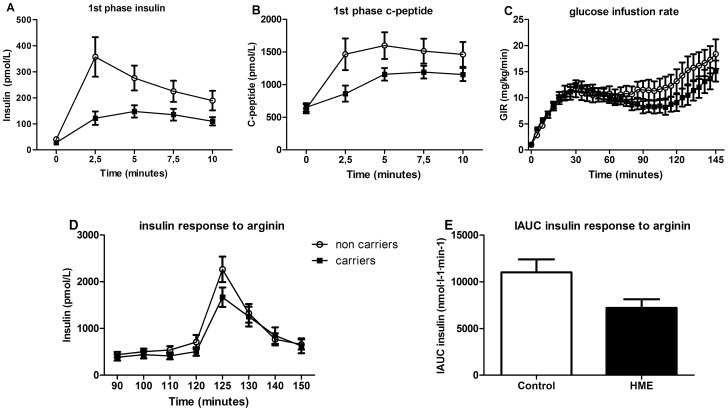
Functional (GSIS) pancreas reserve in HME subjects (closed sq) versus controls (circles). A and B: The first-phase insulin and C-peptide response to a hyperglycaemic clamp was lower in HME subjects compared to controls. C: The glucose infusion rate (GIR), an estimation of the amount of glucose being metabolized, was not different between groups. D and E: Insulin secretion after an intravenous bolus of arginine was lower in carriers vs controls. * p = 0.028.

We noted a trend towards lower plasma insulin levels in HME subjects, thus we subsequently performed a hyperglycemic normoinsulinemic clamp followed by arginine infusion. During the hyperglycemic clamp, first phase insulin response to an intravenous glucose bolus (as determined by incremental AUC) was lower in carriers than control subjects (0.72 [0.46–1.16] vs. 1.53 [0.69–3.36] nmol·l^−1^·min^−1^, p = 0.017) (Fig. 2A). In addition, C-peptide responses were also significantly lower in carriers (3.57 [2.26–5.00] vs. 6.62 [4.48–9.84] nmol·l^−1^·min^−1^ p<0.008) (Fig. 2B). In line with the HOMA data, glucose infusion rates were comparable between groups (iAUC carriers vs. controls 11.1 [8.88–19.00] vs. 14.5 [11.98–23.98] mg·kg^−1^·min^−1^ n.s.) (Fig. 2C), as well as disposition indices (Table 2), suggesting that differences observed in insulin and C-peptide secretion cannot be attributed to differences in insulin tolerance. Following an intravenous arginine bolus to assess maximal insulin reserve, the peak response was significantly impaired in *EXT* carriers compared to controls (iAUC from t = 120: 7.14 [4.22–10.95] vs. 10.32 [7.91–12.70] nmol·l^−1^·min^−1^ p<0.028) (Figs. 2D and E).

**Figure 3:**
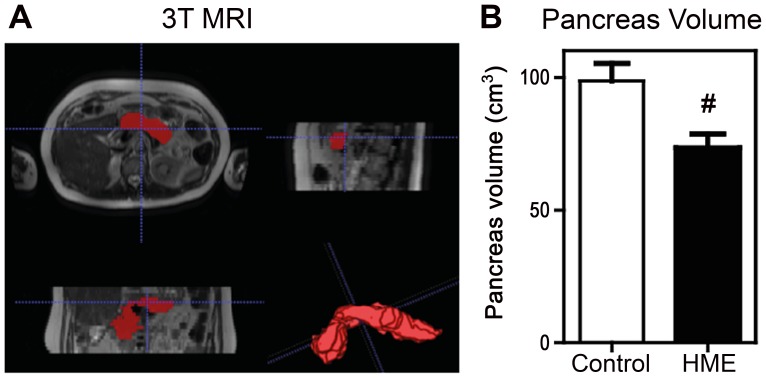
Pancreas volume, assessed with 3T MRI, was smaller in HME subjects than controls (A) Example of axial (top left), sagital (top right) and coronal (bottom left) view and 3D visualization (bottom right) of delineated pancreas. (B) Pancreatic volumes (cm^3^) in HME subjects and controls. # p = 0.04.

To further investigate the decreased beta-cell insulin secretory capacity we set out to detect potential differences in anatomical pancreatic volume in n = 8 *EXT* carriers and n = 8 non-carrier controls (Table 3). Abdominal MRI imaging revealed a significantly smaller pancreatic volume in *EXT* carriers compared to control subjects (72.0±15.8 vs. 96.5±26.0 cm^3^ p = 0.04) (Fig 3).

**Table 3:** Baseline characteristics of participants in MRI.

	Noncarriers	Carriers	P-value
	(*N = 8)*	(*N = 8)*	
Age (years)	40±13	39±9	0.85
Men	3 (37)	3 (37)	
Length (m)	1.72±0.10	1.72±0.10	0.89
Weight	74±9	78±13	0.60
BMI	24.6±1.4	25.6±4.4	0.54
BSA	1.8±0.15	1.9±0.18	0.38

Data are means ± SD, *n (%)*, or median [IQR]. Abbreviations: BMI  =  body mass index; BSA  =  body surface area.

## Discussion

Here we provide the first evidence that carriers of loss-of-function mutations in *EXT* have a distinct perturbation in glucose-insulin homeostasis characterized by an impaired glucose stimulated insulin secretion response as well as a decreased peak-insulin secretory capacity. The latter is accompanied by a significant reduction in total pancreas volume, implying a structural beta-cell defect in carriers of loss-of-function mutations in *EXT*.

*EXT* mutation carriers displayed normal insulin sensitivity during OGTT. However, a trend towards reduction in plasma insulin response during OGTT was observed in these subjects. Using hyperglycemic clamps, we observed that *EXT* carriers were characterized by a reduced first-phase insulin response to hyperglycemia (GSIS) compared to noncarriers matched for age, sex, and BMI. Interestingly, in a cohort of Pima Indians, who are marked by high levels of insulin resistance and obesity, an association between SNPs in *EXT2* and incidence of DM2 was found [23]. Together these findings suggest that HME subjects, being carriers of heterozygous EXT mutations might be at increased risk of developing DM2 when becoming obese, due to their underlying beta-cell defect.

### Functional beta-cell defects in heterozygous carriers of EXT mutations

Our findings elute to either a functional, signalling defect of the beta-cell or a structural diminished β-cell mass. It has been recognized that GSIS reflects the available previously synthesized and stored insulin that can be secreted upon glucose stimulation [31]. After entering the β-cell via GLUT transporters, glucose is modified by glucokinase, the rate-limiting step in glucose sensing. Glycolysis results in ATP production and subsequently the ATP-sensitive potassium channel is closed, followed by membrane depolarization, increased calcium influx via the L-type calcium channel and finally, exocytosis of insulin-containing granules. This first-phase secretory response is augmented by a potassium channel-independent pathway, which is largely responsible for the second-phase insulin response [32].

It has been previously shown that specific inhibition of heparan sulfate synthesis in a mouse model by *Extl3* knock-down results in impaired GSIS [7]. Inline, Takahashi et all showed that treatment of isolated islets with heparinase resulted in decreased insulin secretion upon glucose stimulation, together with decreased expression of *Glut2, Sur1* and *Stx1A*. These data underline the important role for intact heparan sulfate in GIIS [7]. However, the expression of *GLUT*2 in human beta-cells is very low compared to that in mice pancreas [33]. Therefore, the proposed explanation for the reduction in insulin secretory capacity in these models through reduced GLUT2 expression with subsequent attenuated FGF signaling [34] can't be translated 1∶1 to the human situation.

Beta-cell function may also be affected by impaired Hedgehog (Hh) signalling, which has been proposed to play a role in insulin production [35], [36] and beta-cell function [37] throughout life. It was shown that tout-velu (ttv), an EXT1 analog in *Drosophilia*, is required for Hh diffusion [38], thus linking disturbances in heparan sulfate to beta-cell function. In this regard, it has also been reported that Wnt proteins are involved in GSIS in adult mouse islets [39].

### Structural beta-cell defects in heterozygous carriers of EXT mutations

Both the first-phase insulin responses as well as the secretory responses to arginine were significantly impaired in *EXT* carriers. These findings contrast our previous results in subjects carrying a heterozygous mutation in *ABCA1*, showing decreased GSIS with an intact maximal insulin release capacity following arginine [28]. Arginine stimulates insulin secretion by directly inducing membrane depolarization independent of potassium channels and thus largely independent of glucose sensing and glucose metabolism pathways [40], [41]. Our findings suggest that in HME a structural, rather than a functional defect, may lead to decreased GSIS and arginine-insulin responses. MRI based pancreatic volume measurements in our HME subjects indeed lend further support to a structural defect in *EXT* carriers.

### Beta-cell survival

In a recent study it was implicated that HSPG was involved in beta-cell survival, providing a buffer mechanism against reactive oxygen species (ROS) in the murine pancreas [42]. Indeed, it has been previously noted that beta-cell failure precedes the development of impaired glucose tolerance (IGT) in insulin resistant subjects [43] due to ROS induced exhaustion of the normal beta-cell capacity to adjust for increased insulin demand [44]. Thus HSPGs may have several roles in beta-cell homeostasis via either regulation of postnatal islet and pancreas development [7] or protection of the beta-cell against destruction later in life [45]. The inadvertent depletion of pancreas heparan sulfates in *EXT* carriers might render (the already decreased amount of) beta-cells vulnerable for exogenous pathogenic stimuli including obesity and older age. Unfortunately however, at this moment data on development of DM2 in HME patients cohorts of older age are not available, as increased morbidity and mortality due to malignant bone tumours resulted in loss of follow up.

### Insulin signalling in HME carriers

The development of type 2 diabetes is a complex interplay of declining beta-cell function and subsequent development of insulin resistance, influenced by both genetic and environmental factors. In this respect, the elevated levels of osteocalcin found in our patients may play an interesting role. The elevated levels of osteocalcin might reflect altered bone formation in HME subjects, whom are characterized by the development of bony tumors [29]. Although in mouse models, osteocalcin improves insulin sensitivity [46] and beta-cell proliferation [47], translation of these data to our subjects with HME should be done with caution. For example, most epidemiological studies (reviewed in ref [29]) show an inverse correlation between plasma osteocalcin levels and the presence of insulin resistance. Murine studies have suggested that one of the mechanisms by which osteocalcin improves beta-cell function is through increased GLP-1 secretion [48], [49]. As we did not find differences in fasting GLP-1 levels in our subjects, this mechanism does not seem to be responsible for the found increase in beta cell function in our study. Nevertheless, further study on the relation between altered bone metabolism and glucose metabolism in HME subjects is warranted.

### Study limitations

Several issues in our study deserve closer attention. First, based on the small number of available mutation carriers in The Netherlands this precludes analysis regarding individual effects of EXT1 or EXT2 on beta-cell function and size. However, although mutations in either *EXT1* or *EXT2* can result in development of Hereditary Multiple Exostoses, mutations in *EXT1* are associated with a higher disease burden [50], [51]. This is most likely due to an more pronounced biological function for EXT1 albeit that EXT2 is required to allow the proper function of EXT1 [52].

Second, a reduced pancreas volume could be accompanied by an initial protective increase in islet proliferation. In the present study, we cannot rule out this possibility, however, our functional data regarding the impaired maximum insulin release upon the arginine bolus in HME subjects do not support this hypothesis.

Finally, as large clinical cohorts of well genotyped HME subjects are currently not available, further studies are needed to address the question whether a similar mechanism of decreased pancreas volume might partially underly the genetic association between *EXT* and defective insulin secretion in HME subjects with differences in BMI.

Nevertheless, we now provide the first evidence on the relation between genetic defects in heparan sulfate synthesis and decreased pancreas anatomic volume with ensuing impaired beta-cell reserve capacity in carriers of loss-of-function mutations in *EXT*.

## Supporting Information

S1 Table **Mutations in the** ***EXT1*** **gene in our cohort.**
(DOC)

S2 Table **Mutations in the** ***EXT2*** **gene in our cohort.**
(DOC)

S3 Table **Baseline characteristics of study subjects in OGTT.**
(DOC)

S4 Table **Baseline characteristics of study subjects in clamp.**
(DOC)

S1 File **Supporting information file containing data for baseline characteristics.**
(SAV)

S2 File **Supporting information file containing data for OGTT.**
(SAV)

S3 File **Supporting information file containing data for Clamp.**
(SAV)

S4 File **Supporting information file containing data for MRI.**
(SAV)
