# Supplementary material for: Carriers of Loss-of-Function Mutations in EXT Display Impaired Pancreatic Beta-Cell Reserve Due to Smaller Pancreas Volume
Source: PLoS One. 2014 Dec 26;9(12):e115662. doi: 10.1371/journal.pone.0115662 (PMC4277348; doi:10.1371/journal.pone.0115662)
Supplement: S1 Table — Mutations in the EXT1 gene in our cohort. (DOC) [file pone.0115662.s001.doc]

**Table S1: Mutations in the *EXT1* gene in our cohort**

| **Exon** | **cDNA change** | **Protein** |
| --- | --- | --- |
| 1 | c.864del. | Asn288fs |
| 1 | c.1384del, | Leu462fs |
| 10 | c.1884-1G>C |  |
| 1 | c.393C>A, | Tyr131x |
| 2 | c.1031C>T, | Ser344Phe |
| 6 | c.1431dup | Ser478fs |
| 1 | c.138del | Leu46fs |
| 7, 8 | Deletion exon 7 |  |
|  | and 8 |  |
| 1 | c.1019G>A | Arg340His |
| 1 | c. 679del | Arg227fs |
| 1 | c.766_773del | p.Ile256Serfs*30 |
