# Supplementary material for: Carriers of Loss-of-Function Mutations in EXT Display Impaired Pancreatic Beta-Cell Reserve Due to Smaller Pancreas Volume
Source: PLoS One. 2014 Dec 26;9(12):e115662. doi: 10.1371/journal.pone.0115662 (PMC4277348; doi:10.1371/journal.pone.0115662)
Supplement: S2 Table — Mutations in the EXT2 gene in our cohort. (DOC) [file pone.0115662.s002.doc]

**Table S2: Mutations in the *EXT*2 gene in our cohort**

| **Exon** | **cDNA change** | **Protein change** |
| --- | --- | --- |
| 2 | dup | Val68fs |
| 2 | 1000T>C | Cys334Arg |
| 7 | 1173+1G>A |  |
| 7 | 1080-2A>G |  |
