# Supplementary material for: Carriers of Loss-of-Function Mutations in EXT Display Impaired Pancreatic Beta-Cell Reserve Due to Smaller Pancreas Volume
Source: PLoS One. 2014 Dec 26;9(12):e115662. doi: 10.1371/journal.pone.0115662 (PMC4277348; doi:10.1371/journal.pone.0115662)
Supplement: S3 Table — Baseline characteristics of study subjects in OGTT. (DOC) [file pone.0115662.s003.doc]

**Table S3: Baseline characteristics of study subjects in OGTT**

|  | **Noncarriers** | **Carriers** | **P-value** |
| --- | --- | --- | --- |
|  | **(N=9)** | **(N=11)** |  |
| Age (years) | 44±13 | 40±10 | 0.35 |
| Men | 4 (44) | 4 (36) |  |
| BMI | 25.6±3.4 | 25.8±4.9 | 0.89 |
| BSA | 1.9±0.20 | 1.9±0.18 | 0.56 |
| Cholesterol (mmol/l) |  |  |  |
| Total | 5.43±1.40 | 5.07±0.97 | 0.51 |
| LDL | 3.31±1.43 | 3.13±0.87 | 0.72 |
| HDL | 1.41±0.42 | 1.51±0.47 | 0.62 |
| Triglycerides (mmol/l) | 0.92[0.68-1.38] | 0.87[0.56-1.32] | 1.00 |
| Fasting glucose (mmol/l) | 5.0±0.45 | 4.8±0.57 | 0.50 |
| Hba1c |  |  |  |
| mmol/mol | 35±4.5 | 36±3.9 | 0.70 |
| % | 5.4±0.40 | 5.4±0.35 | 0.75 |
| Fasting insulin (pmol/l) | 51±44 | 48±35 | 0.83 |
| GLP-1 (pmol/l) | 5.2[5.2-5.2] | 5.2[5.2-5.6] | 0.65 |
| Fasting FFA (mmol/l) | 0.46[0.32-0.88] | 0.64[0.53-0.77] | 0.29 |
| Osteocalcin (mmol/L) | 1,6±0,8 | 2,9±0,9 | 0.008 |
| Fecal Elastase (ug/g) | >500 | >500 |  |

Data are means ± SD, *n (%),* or median [IQR]. Abbreviations: BMI = Body Mass Index; BSA = Body Surface Area; LDL = Low Density Lipoprotein. HDL = High Density Lipoprotein.
