# Supplementary material for: Carriers of Loss-of-Function Mutations in EXT Display Impaired Pancreatic Beta-Cell Reserve Due to Smaller Pancreas Volume
Source: PLoS One. 2014 Dec 26;9(12):e115662. doi: 10.1371/journal.pone.0115662 (PMC4277348; doi:10.1371/journal.pone.0115662)
Supplement: S4 Table — Baseline characteristics of study subjects in clamp. (DOC) [file pone.0115662.s004.doc]

**Table S4: Baseline characteristics of study subjects in clamp**

|  | **Noncarriers** | **Carriers** | **P-value** |
| --- | --- | --- | --- |
|  | **(N=12)** | **(N=14)** |  |
| Age (years) | 50±11 | 38±10 | 0.005 |
| Men | 8 (40) | 7 (30) |  |
| BMI | 25.5±3.8 | 25.5±4.4 | 0.97 |
| BSA | 1.9±0.23 | 1.9±0.19 | 0.36 |
| Cholesterol (mmol/l) |  |  |  |
| Total | 5.04±1.24 | 4.89±1.32 | 0.77 |
| LDL | 3.13±0.98 | 3.13±1.22 | 0.99 |
| HDL | 1.55±0.45 | 1.17±0.25 | 0.01 |
| Triglycerides (mmol/l) | 0.92[0.68-1.38] | 0.87[0.56-1.32] | 0.96 |
| Fasting glucose (mmol/l) | 4.8±0.64 | 4.7±0.40 | 0.91 |
| Hba1c |  |  |  |
| mmol/mol | 37±3.2 | 34±3.2 | 0.13 |
| % | 5.5±0.28 | 5.3±0.28 | 0.14 |
| Fasting insulin (pmol/l) | 44±21 | 30±18 | 0.08 |

Data are means ± SD, *n (%),* or median [IQR]. Abbreviations: BMI = Body Mass Index; BSA = Body Surface Area; LDL = Low Density Lipoprotein. HDL = High Density Lipoprotein.
